# Dialysate cell-free mitochondrial DNA fragments as a marker of intraperitoneal inflammation and peritoneal solute transport rate in peritoneal dialysis

**DOI:** 10.1186/s12882-019-1284-3

**Published:** 2019-04-11

**Authors:** Xishao Xie, Junni Wang, Shilong Xiang, Zhimin Chen, Xiaohui Zhang, Jianghua Chen

**Affiliations:** The Kidney Disease Center, The First Affiliated Hospital, College of Medicine, Zhejiang University, 79 Qingchun Rd, Hangzhou, Zhejiang 310003 People’s Republic of China

**Keywords:** Mitochondrial DNA, Inflammation, Peritoneal solute transport rate, Peritoneal dialysis

## Abstract

**Background:**

Mitochondrial DNA (mtDNA) released into extracellular subsequent to cell injury and death can promote inflammation in patients and animal models. However, the effects of peritoneal dialysate cell-free mtDNA on intraperitoneal inflammation and peritoneal solute transport rate (PSTR) in peritoneal dialysis (PD) patients remain unclear.

**Methods:**

We select the incident patients who began PD therapy between January 1, 2009, and December 30, 2010. Peritoneal dialysate was collected at the time of peritoneal equilibration test. The cell-free mtDNA, IL-6, IL-17A, TNF-α and IFN-γ were measured. All patients were followed till December 2017. The results were compared with PSTR and patient survival.

**Results:**

One hundred and eighty-nine patients were included in the study. The average age was 47.1 ± 13.5 years, 55.6% of the patients were males. The average PSTR was 0.66 ± 0.12, the median dialysate mtDNA levels were 4325 copies/ul. The median concentrations of IL-6, IL-17A, TNF-α and IFN-γ were 25.9, 10.8, 25.8 and 17.9 pg/ml, respectively. We found that dialysate mtDNA was significantly correlated with PSTR (*r* = 0.461, *P* < 0.001), IL-6 (*r* = 0.568, *P* < 0.001), TNF-α (*r* = 0.454, *P* < 0.001) and IFN-γ (*r* = 0.203, *P* = 0.005). After adjustment for multiple covariates, dialysate mtDNA levels were independently correlated with IL-6 and PSTR. Dialysate mtDNA levels were not associated with patient survival.

**Conclusions:**

We found that dialysate mtDNA levels correlated with the degree of intraperitoneal inflammatory status in PD patients. Peritoneal effluent mtDNA was an independent determinant of PSTR but did not affect patient survival.

**Electronic supplementary material:**

The online version of this article (10.1186/s12882-019-1284-3) contains supplementary material, which is available to authorized users.

## Background

The structural and functional integrity of peritoneal membrane was the essential for peritoneal dialysis (PD). Peritoneal membrane function varies widely among individuals, and incident PD patients with a high peritoneal solute transport rate (PSTR) have been linked to increased mortality and technique failure [1–3]. The associations of local, chronic peritoneal inflammation with PSTR have been reported in prior studies [4–6], and the multicenter GLOBAL study has demonstrated that the intraperitoneal inflammation (dialysate IL-6) is the most reliable predictor of PSTR [7]. However, the cause of chronic intarperitoneal inflammation is not completed understood.

Besides of exogenous pathogen-associated molecular patterns (PAMPs), such as lipopolysaccharides (LPS), peptidoglycan and bacterial DNA, many studies have noted that various endogenous damage-associated molecular patterns (DAMPs) could activate the immune system in a non-infectious state [8, 9]. Mitochondria was evolved from saprophytic bacteria to endosymbionts to organelles, and Mitochondrial DNA (mtDNA) shows similarities to bacterial DNA [10]. mtDNA released into the extracellular space subsequent to cell injury and death acts as DAMPs could induce inflammatory response through Toll-like receptor 9 (TLR9) pathway [11, 12]. Increased plasma mtDNA levels have been found in patients with trauma, sepsis and some autoimmune diseases, and were associated with the inflammation status and survival [13–16]. In PD patients, the PD catheters, long-term exposures to PD solutions and repeated peritonitis episodes could induce cell injury and death, which would lead to an abundant release of mtDNA into local intraperitoneal cavity. The purpose of the present study was to explore the relationships between mtDNA in peritoneal dialysate and intraperitoneal inflammation, PSTR and survival in PD patients.

## Methods

### Study population

All ESRD patients who began PD therapy at the First Affiliated Hospital of Zhejiang University in China between January 2009 and December 2010 were considered eligible for this study. Exclusion criteria were as follows: an age below 18 or above 80 years, a follow-up of less than 6 months, without peritoneal permeability test (PET) within 6 months after the initiation of PD therapy, peritonitis in the previous 1 month before PET. This study was approved by the Clinical Research Ethics Committee of the First Affiliated Hospital of Zhejiang University and performed in compliance with the Declaration of Helsinki. All participants provided informed consent before enrollment.

Baseline clinical data including age, gender cause of ESRD, body mass index (BMI), comorbid conditions, were recorded. The Charlson comorbidity index (CCI) was used to calculate a comorbidity score [17]. The collected biochemical data at the time of PET included hemoglobin, serum albumin and high sensitive C-reactive protein. All the biochemical parameters were assessed in the center laboratory of our hospital. PD-related data including dialysis regimen and dose were recorded. Standard PET was performed by the method of Twardowski [18], and peritoneal solute transport rate (PSTR: dialysate-to-plasma creatinine ratio) and ultrafiltration capacity at 4 h with 2.5% glucose were calculated. The glucose exposure was measured as total grams of glucose within the daily dialysate. Residual renal function was calculated as the mean of the urea and creatinine clearance. Total Kt/Vurea and peritoneal Kt/Vurea were calculated based on 24-h urine and dialysate collection using PD Adequest software (Baxter Healthcare).

### Dialysate mitochondrial DNA and cytokines level

Dialysate sample was collected from a 4-h PET. The sample was centrifuged for at 1500 g at 4 °C for 10 min, and the supernatant was saved and kept frozen at − 80 °C until analysis. DNA was extracted from 200ul of dialysate supernatant using QIAamp DNA Mini Kit and Blood Mini Kit (Qiagen). The concentration of mtDNA was measured with mitochondrial cytochrome B (Cyto B, forwad 5′-ATGACCCCAATACGCAAAAT-3′, reverse 5′-CGAAGTTTCATCATGCGGAG-3′) genes by real-time quantitative polymerase chain reaction (qPCR) assay (Applied Biosystems). The qPCR reacton mixture consisted of 10 ul 2 × SYBR Green Master Mix (Applied Biosystems), 0.5 ul forward primer (5 uM), 0.5 ul reverse primer (5 uM) of DNA and 4 ul nuclease-free water. qPCR was carried out as follows: a first denaturation step at 95 °C, and followed by 40 cycles at 95 °C for 30s, 53 °C for 30s and 72 °C for 30s. The absolute values of mtDNA were determined by calculation from a stand curve using a cloned plasmid DNA (Additional file 1: Figure S1), as has been described previously [19, 20]. The mtDNA levels in dialysate were expressed in copies/ul.

Dialysate supernatant IL-6, IL-17A, TNF-α and IFN-γ levels were assessed by ELISA (R&D System) following the protocol recommended by the manufacturer.

### Clinical follow-up

The primary outcome measure was patient survival. Death and transfer to hemodialysis were regarded as composite endpoint events. Kidney transplantation was regarded as the censored event. All patients were followed till December 31, 2017.

### Statistical analysis

Continuous variables are presented as means ± SD or the medians with the interquartile ranges, and categorical variables are presented as frequencies with percentages. Differences between groups were compared with independent t test, Kruskal-Wallis test, chi-square tests, or one-way ANOVA test as appropriate.

Dialysate mtDNA and cytokines were log_10_ transformed and the correlations between mtDNA and cytokines and Kt/Vurea were assessed by Pearson’s correlation analysis. General linear regression model was used to identify the predictors of IL-6 and PSTR. Covariates with *P*-value < 0.1 in univariate models were selected for multivariate regression models.

Survival was analyzed using the Kaplan-Meier method, and the groups were compared using a log-rank test. The patients were divided into the following 3 groups based on the mtDNA level tertiles: tertile 1 (< 2630 copies/ul), tertile 2 (2630~6300 copies/ul) and tertile 3 (> 6300 copies/ul). The Cox proportional hazards regression model was used to identify independent predictors of patient survival after adjusting for potential confounders. All statistical analyses were performed using SPSS version 22.0 (SPSS Inc.). A value of *P* < 0.05 was considered statistically significant.

## Results

### Patient characteristics

**Fig. 1:**
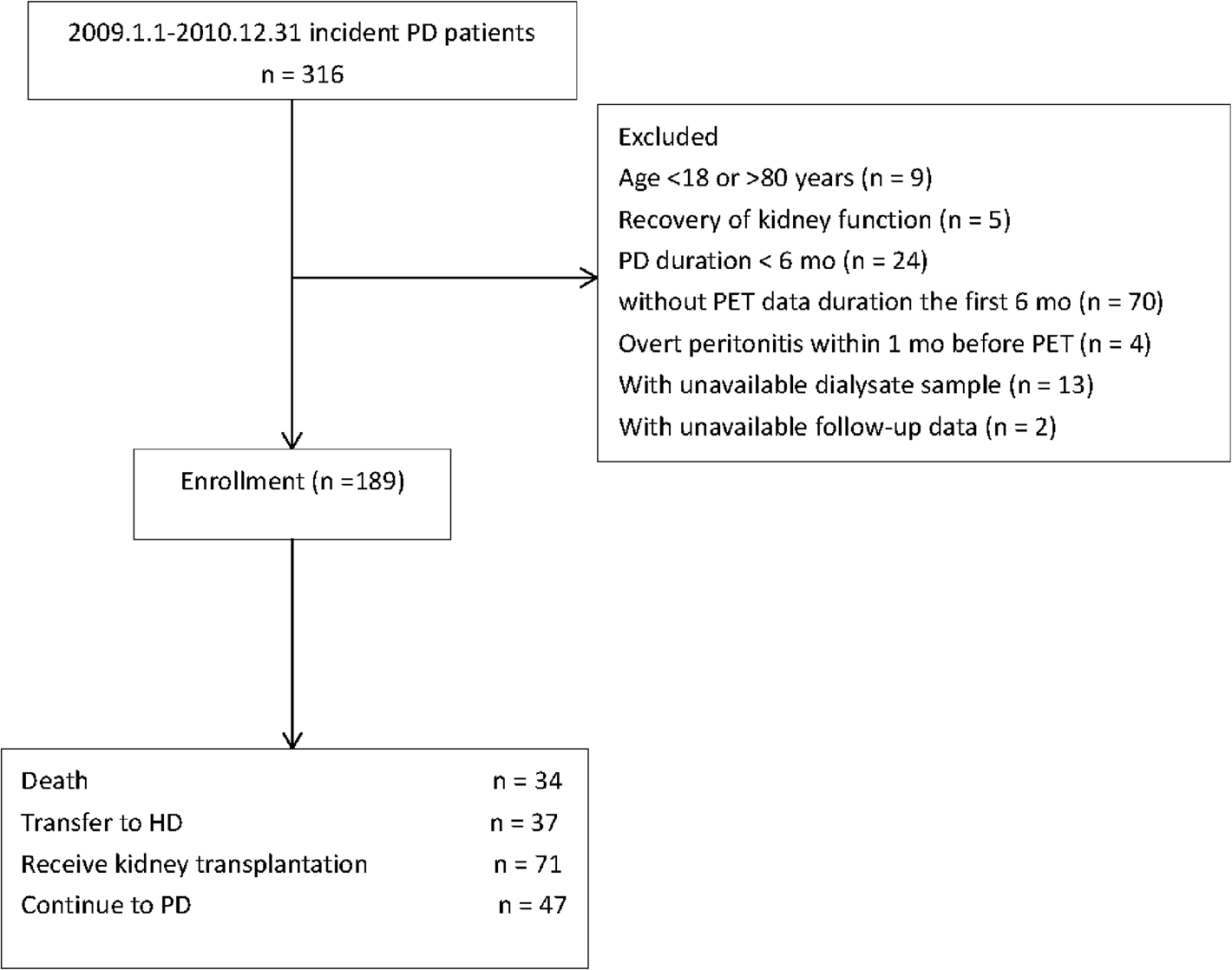
Flow chart of the participants in the study cohort

A total of 189 patients were included in the final analysis (Fig. 1). The baseline clinical and biochemical data are summarized in Table 1. The mean age of the cohort was 47.1 ± 13.5 years, 55.6% of the patients were male. The median time of PET was 2.7 months after initiation of PD, and the mean PSTR was 0.66 ± 0.12. The median level of dialysate mtDNA was 4325 copies/ul. For incident patient, the timing of the initial PET had no significant effect on the level of mtDNA (< 3 mo vs. > 3 mo, *P* = 0.194, Additional file 1: Figure S2). The median levels of dialysate IL-6, IL-17A, TNF-α and IFN-γ was 25.9 pg/mL, 10.8 pg/mL, 25.8 pg/mL and 17.9 pg/mL, respectively (Table 2).

**Table 1:** Study population characteristics

	Total (189)
Sex (male: female)	105:84
Age (years)	47.1 ± 13.5
Primary renal disease	
Chronic glomerulonephritis (%)	133 (70.4)
Diabetic nephropathy (%)	10 (5.3)
Hypertensive nephrosclerosis (%)	21 (11.1)
Obstructive nephropathy (%)	8 (4.2)
Polycystic kidneys (%)	4 (2.1)
Other/unknown (%)	13 (6.9)
BMI (kg/m^2^)	21.0 ± 3.0
Diabetes (%)	23 (12.2)
CCI	3.0 ± 1.3
Hemoglobin (g/dL)	8.2 ± 1.6
Serum albumin (g/L)	38.1 ± 5.4
hs-CRP (mg/dL)	2.4 (1.1–4.1)
Residual GFR (ml/min)	7.3 ± 2.5
urine volume (ml/24 h)	900 (500–1300)
Ultrafiltration volume (ml/24)	250 (100–500)
Total dialysate volume (L/24 h)	5.5 ± 1.4
Dialysate glucose exposure (g/24 h)	90 (60–110)
2.5% glucose solution use (%)	97 (51.3)
Peritoneal transport status	
High (%)	18 (9.5)
High average (%)	57 (30.2)
Low average (%)	57 (30.2)
Low (%)	20 (10.6)
PSTR(4 h)	0.66 ± 0.12
Time of PET (months)	2.7 (1.9–4.7)
Peritoneal Kt/Vurea	1.11 ± 0.45
Total Kt/Vurea	2.15 ± 0.56
Follow-up (months)	41.9 (22.4–63.5)

Abbreviations: BMI, body mass index; CCI, Charlson comorbidity index; hs-CRP, high sensitivity C-reactive protein; Residual GFR, residual glomerular filtration rate; PSTR, Peritoneal solute transport rate; PET, Peritoneal equilibration test

**Table 2:** Dialysate mtDNA and cytokine concentrations

	Total (*n* = 189)
mtDNA (copies/uL)	4325 (1967–8766)
IL-6 (pg/mL)	25.9 (18.8–32.2)
IL-17A (pg/mL)	10.8 (9.9–11.8)
TNF-α (pg/mL)	25.8 (18.9–28.4)
IFN-γ (pg/mL)	17.9 (15.2–20.5)

Abbreviations: mtDNA, Mitochondrial DNA

### Correlation between mtDNA, cytokines and PSTR

**Fig. 2:**
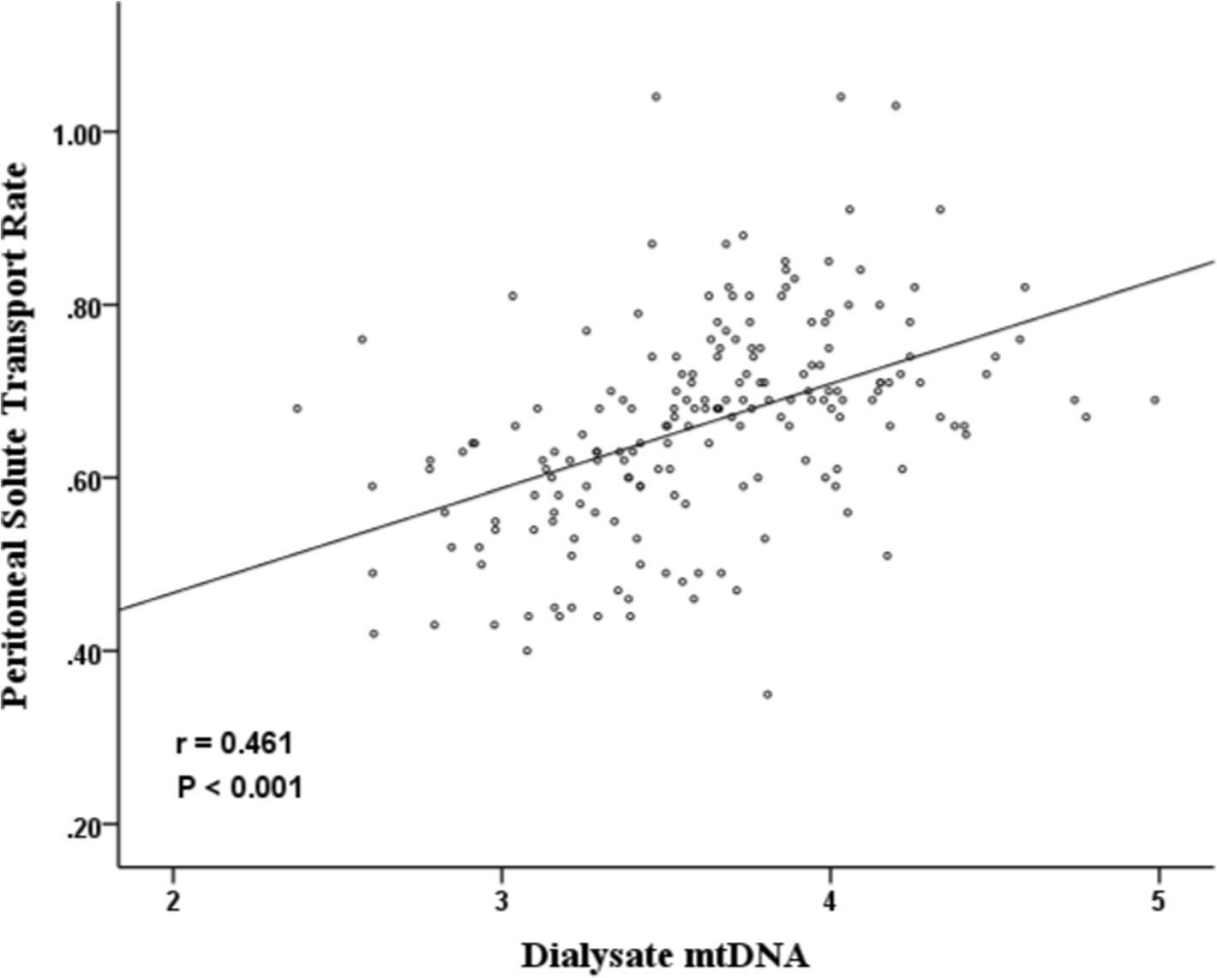
Scatterplot of PSTR with dialysate mtDNA. mtDNA coefficient was per log_10_ changes in concentrations. A positive correlation between dialysate mtDNA and PSTR was observed

Results of correlation analysis between mtDNA and cytokines are shown in Table 3. Dialysate mtDNA levels were significantly correlated with dialysate IL-6 (*r* = 0.568, *P* < 0.001), TNF-α (*r* = 0.454, *P* < 0.001) and IFN-γ (*r* = 0.203, *P* = 0.005) concentrations. No relationship between dialysate mtDNA and IL-17A was observed (*r* = 0.027, *P* = 0.710). Dialysate mtDNA level had significant correlation with PSTR (*r* = 0.461, *P* < 0.001, Fig. 2). PSTR also significantly correlated with IL-6, TNF-α and IFN-γ (Additional file 1: Figure S3).

**Table 3:** Correlation analysis between mtDNA and cytokines

		mtDNA	IL-6	IL-17	TNF-α
IL-6	*r*	0.568			
	*p*	< 0.001			
IL-17A	*r*	−0.004	0.185		
	*p*	0.959	0.011		
TNF-α	*r*	0.454	0.580	0.065	
	*p*	< 0.001	< 0.001	0.372	
IFN-γ	*r*	0.203	0.390	0.279	0.220
	*p*	0.005	< 0.001	< 0.001	0.002

mtDNA and cytokine coefficients are per log_10_ changes in concentrations

### Factors associated with IL-6 and PSTR

Results of the multivariable general linear regression models showing the predictors of IL-6 and PSTR are summarized in Tables 4 and 5 (Results of univariate analysis was shown in Additional file 1: Table S1 and S2). Dialysate mtDNA was significantly associated with IL-6 after adjusting for other confounders including age, sex, serum albumin, IL-17A, TNF-α and IFN-γ, all of which had significant associations. mtDNA, IL-6, sex, and the use of 2.5% glucose solution was independently associated with PSTR.

**Table 4:** Predictors of IL-6

Variable	Coefficient (95% CI)	*P* Value
Age (per 10 years)	0.017 (0.005 to 0.029)	0.004
Sex (Female)	−0.032 (−0.064 to 0)	0.049
BMI	−0.003 (− 0.008 to 0.002)	0.265
Serum albumin (per 1 g/dl)	− 0.034 (− 0.064 to − 0.005)	0.021
mtDNA	0.118 (0.079 to 0.157)	< 0.001
IL-17A	0.276 (0.030 to 0.521)	0.028
TNF-α	0.429 (0.305 to 0.554)	< 0.001
IFN-γ	0.318 (0.158 to 0.478)	< 0.001

Abbreviations: BMI, body mass index; mtDNA and cytokine coefficients are per log_10_ changes in concentrations

**Table 5:** Predictors of PSTR

Variable	Coefficient (95% CI)	*P* Value
Sex (Female)	−0.295 (− 0.575 to − 0.015)	0.039
2.5% glucose solution use	0.761 (0.257 to 1.264)	0.003
Dialysate glucose exposure (per 10 g)	−0.118 (− 0.289 to 0.053)	0.175
Dialysate volume (per 1 L)	0.296 (−0.025 to 0.617)	0.071
mtDNA	0.703 (0.344 to 1.062)	< 0.001
IL-6	2.065 (0.896 to 3.234)	0.001
TNF-α	−0.108 (−1.285 to 1.070)	0.858
IFN-γ	1.377 (−0.007 to 2.762)	0.051

mtDNA and cytokine coefficients are per log_10_ changes in concentrations

### Associations of mtDNA levels with Kt/Vurea and patient survival

**Fig. 3:**
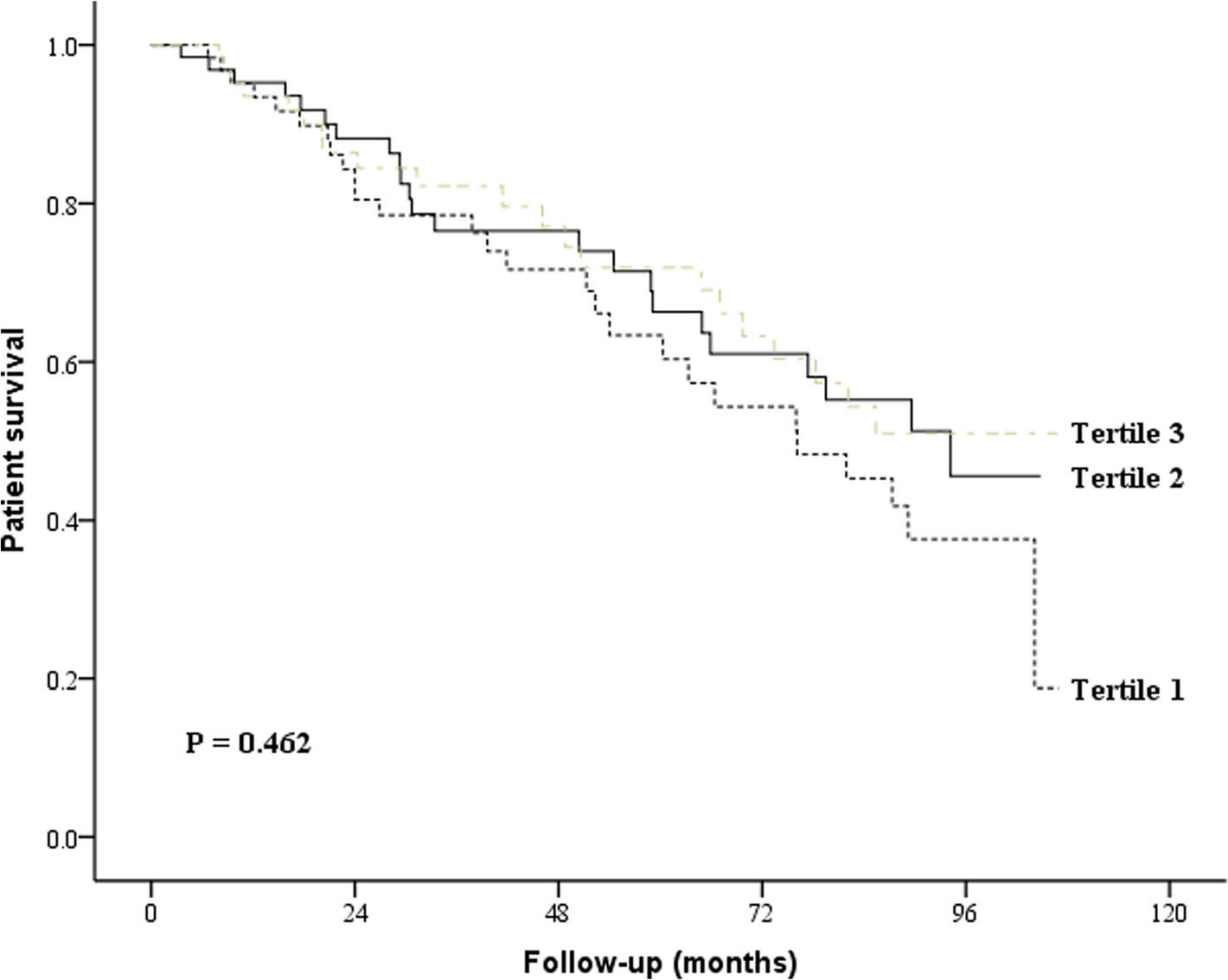
Kaplan-Meier survival curves for the patients with different mtDNA levels (log-rank test, *P = 0.462*). Death and transfer to hemodialysis were regarded as composite endpoint events

Patients contributed a total of 9676.8 months of follow-up. During the study period, 34 (18.0%) patients died, 37 (19.6%) were transferred to HD, and 71 (37.6%) patients underwent kidney transplantation. No significant relationship between baseline dialysate mtDNA and peritoneal Kt/Vurea was observed (r = − 0.26, *P* = 0.719), and there was no significant difference in peritoneal Kt/Vurea and total Kt/Vurea was observed between the three groups during during the study period. Similar results were also observed between dialysate mtDNA and total Kt/Vurea (Additional file 1: Figure S4). At 60 months, the overall patient survival was 61.2, 66.3 and 69.3% for dialysate mtDNA level tertile 1, tertile 2 and tertile 3, respectively. There was no significant difference in patient survival between the three groups(*P* = 0.462, Fig. 3). According to the multivariable Cox proportional hazards model analysis, age, Charlson comorbidity score, 24 h urine volume, serum albumin and hemoglobin were significantly associated with patient survival, whereas dialysate mtDNA had no independent effect (Additional file 1: Table S3).

## Discussion

In the present study, we found that dialysate mtDNA level was significantly associated with the degree of local intraperitoneal IL-6 level, and was a predictor of PSTR. No significantly correlations were founded between dialysate mtDNA and survival in PD patients.

Increasing evidence from studies supports that mtDNA released from injured cells into extracellular space acts as a DAMP and plays an important role in inflammatory responses [11, 21–23]. mtDNA contains unmethylated CpG DNA motifs similar to bacterial DNA, which could bind to TLR9 and activate the downstream inflammatory cascades. Zhang et al. showed that injection of mtDNA could induce proinflammatory cytokines accumulation and acute lung injury with infiltration of polymorphonuclear neutrophils in mice [24], suggesting the direct roles of mtDNA on inflammation and tissue injuries. Tsuji et al. also demonstrated that mtDNA could activate TLR9 and contributes to cytokines production and acute kidney injury during polymicrobial sepsis [25]. Clinical studies have reported that cell-free mtDNA levels in plasma were elevated in several diseases, such as trauma [13, 26], bacterial meningitis [27], sepsis [28, 29], myocardial infarction [30, 31], and maintenance dialysis [32, 33], and were associated with systemic inflammation and mortality.

Although the chronic local intraperitoneal inflammation in PD patients has been demonstrated in prior studies [5, 6, 34], the source and mechanism of local inflammation has not been completely understood. In PD patients, mtDNA could be released to peritoneal cavity in response to repeated exposures to conventional PD solutions and peritonitis episodes. We hypothesize that mtDNA was a pro-inflammatory factor in peritoneal dialysis and we found that dialysate mtDNA was positively correlated with IL-6, TNF-α and IFN-γ, and dialysate mtDNA was a strong predictor of the degree of intraperitoneal inflammation after adjusting for other confounders. To our best knowledge, this is the first study to describe the role of mtDNA in intraperitoneal inflammation in PD patients. Another important observation from our study was the significant association of dialysate mtDNA levels and PSTR. Studies have reported that local subclinical inflammation contributes to progressive peritoneal injury and changes in PSTR [35–37]. The GLOBAL study demonstrated that the local production of IL-6 was the strongest known factor correlated with variability in PSTR in incident PD patients [7], and the balANZ study showed that the increased dialysate IL-6 concentration were able to predict the change in PSTR with longer PD duration [5]. In addition to dialysate IL-6, our study also found that dialysate mtDNA levels were positively associated with PSTR independent of IL-6, the dialysate mtDNA levels in patients with high or high average PSTR were significantly higher than patients with low or low average PSTR (*P* < 0.001). Recent studies showed that extracellular mtDNA could activate endothelial cells, promote adhesion of neutrophils to the endothelium and subsequently increase endothelial permeability [38]. These may partly explain the direct role of dialysate mtDNA on PSTR in PD patient, but the underlying mechanism and pathway needed to be further studied.

The GLOBAL study showed that the systemic inflammation (IL-6 and TNF-α) was the independent predictor of survival, whereas intraperitoneal inflammation and PSTR had no effect [7]. Our results were consistent with the GLOBAL study, in addition to dialysate cytokines levels and PSTR, no correlation between dialysate mtDNA and Kt/Vurea or patient survival were observed in our study. Szeto et al. recently reported that the plasma cell-free mtDNA was a strong predictor of cardiovascular events and need of hospitalization in PD patients [32]. We haven’t examined the plasma mtDNA levels, and more studies were needed to identify the relationships between plasma mtDNA levels and patient survival.

This study has several limitations. Firstly, it was a retrospective study based on the assay of archive dialysate samples from a single center, therefore, our findings only reveal associations and not causation due to the observational nature of the study. Secondly, Long-term storage of dialysate samples may inevitably lead to different degree of changes in mtDNA and cytokines levels, which may result in some bias. Thirdly, we have not examined the changes of dialysate mtDNA level with time, the influence of dynamic changes of dialysate mtDNA level during the observation period on intraperitoneal inflammatory status and PSTR remain unclear.

## Conclusion

In conclusion, we showed that dialysate cell-free mtDNA level was significantly associated with the degree of local intraperitoneal IL-6 level during the first PET, and was a strong predictor of PSTR in PD patients, but not associated with survival. Further studies are needed to identify the underling mechanisms and investigate whether mtDNA can serve as a potential new therapeutic target for reducing the intraperitoneal inflammation and improving the peritoneal membrane function.

## Additional file

Additional file 1: **Figure S1.** Standard curve and dissociation curve from cloned plasmid DNA. **Figure S2.** Dialysate cell-free mtDNA levels of PD patients grouped by the time of the initial PET (< 3 mo vs. > 3 mo). **Figure S3.** Scatterplot of PSTR with dialysate cytokines. Cytokines coefficient was per log_10_ changes in concentrations. **Figure S4.** Changes of peritoneal Kt/Vurea and total Kt/Vurea during the follow up. **Table S1**. Predictors of IL-6 (univariate analysis). **Table S2**. Predictors of PSTR (univariate analysis). **Table S3.** Risk factors of mortality (multivariable Cox regression model). (DOCX 377 kb)
