# Supplementary material for: Dialysate cell-free mitochondrial DNA fragments as a marker of intraperitoneal inflammation and peritoneal solute transport rate in peritoneal dialysis
Source: BMC Nephrol. 2019 Apr 11;20:128. doi: 10.1186/s12882-019-1284-3 (PMC6458606; doi:10.1186/s12882-019-1284-3)
Supplement: Supplementary file 1 — Figure S1. Standard curve and dissociation curve from cloned plasmid DNA. Figure S2. Dialysate cell-free mtDNA levels of PD patients grouped by the time of the initial PET (< 3 mo vs. > 3 mo). Figure S3. Scatterplot of PSTR with dialysate cytokines. Cytokines coefficient was per log10 changes in concentrations. Figure S4. Changes of peritoneal Kt/Vurea and total Kt/Vurea during the follow up. Table S1. Predictors of IL-6 (univariate analysis). Table S2. Predictors of PSTR (univariate analysis). Table S3. Risk factors of mortality (multivariable Cox regression model). (DOCX 377 kb) [file 12882_2019_1284_MOESM1_ESM.docx]

**Additional file 1**

**Figure S1.** Standard curve and dissociation curve from cloned plasmid DNA.


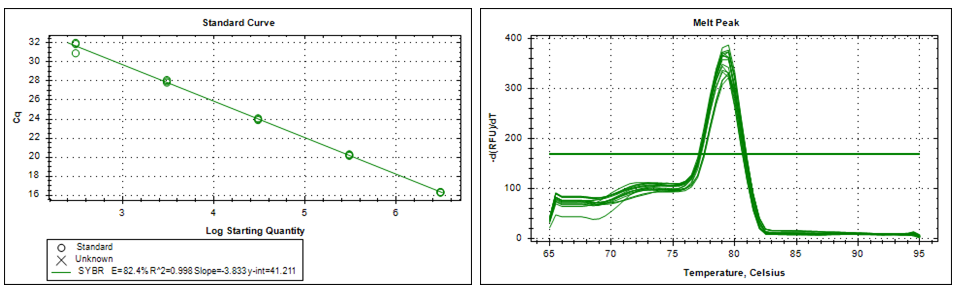


**Figure S2.** Dialysate cell-free mtDNA levels of PD patients grouped by the time of the initial PET (< 3 mo vs. > 3 mo).


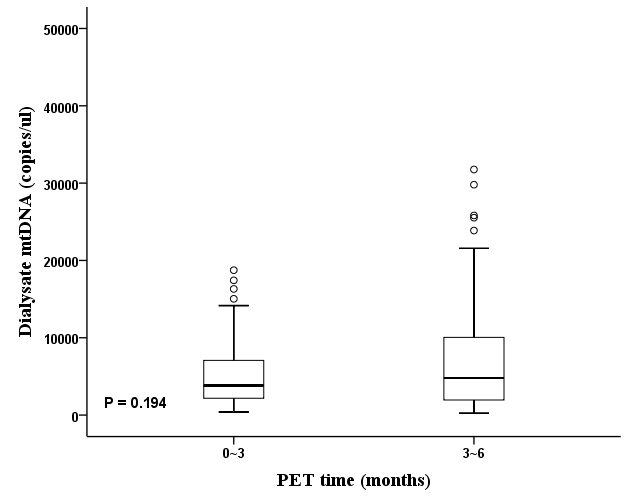


**Figure S3.** Scatterplot of PSTR with dialysate cytokines. Cytokines coefficient was per log_10_ changes in concentrations.


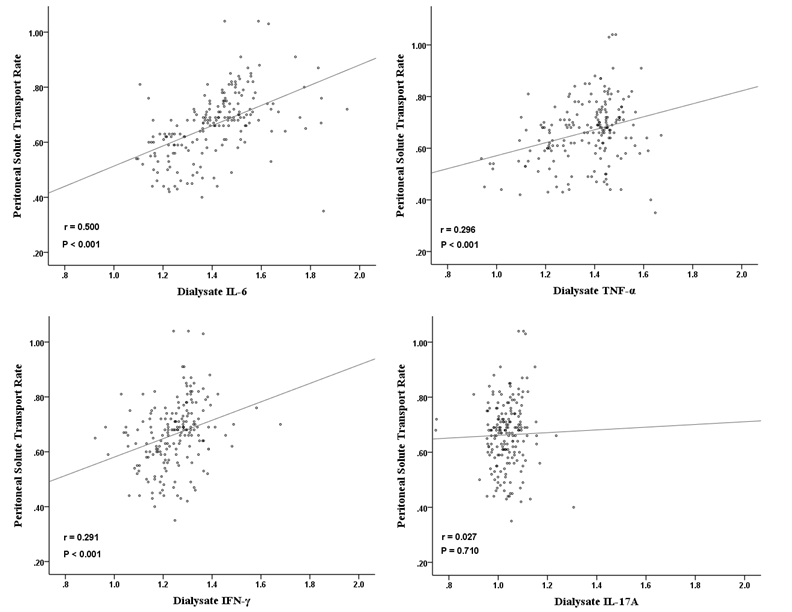


**Figure S4.** Changes of peritoneal Kt/Vurea and total Kt/Vurea during the follow up.


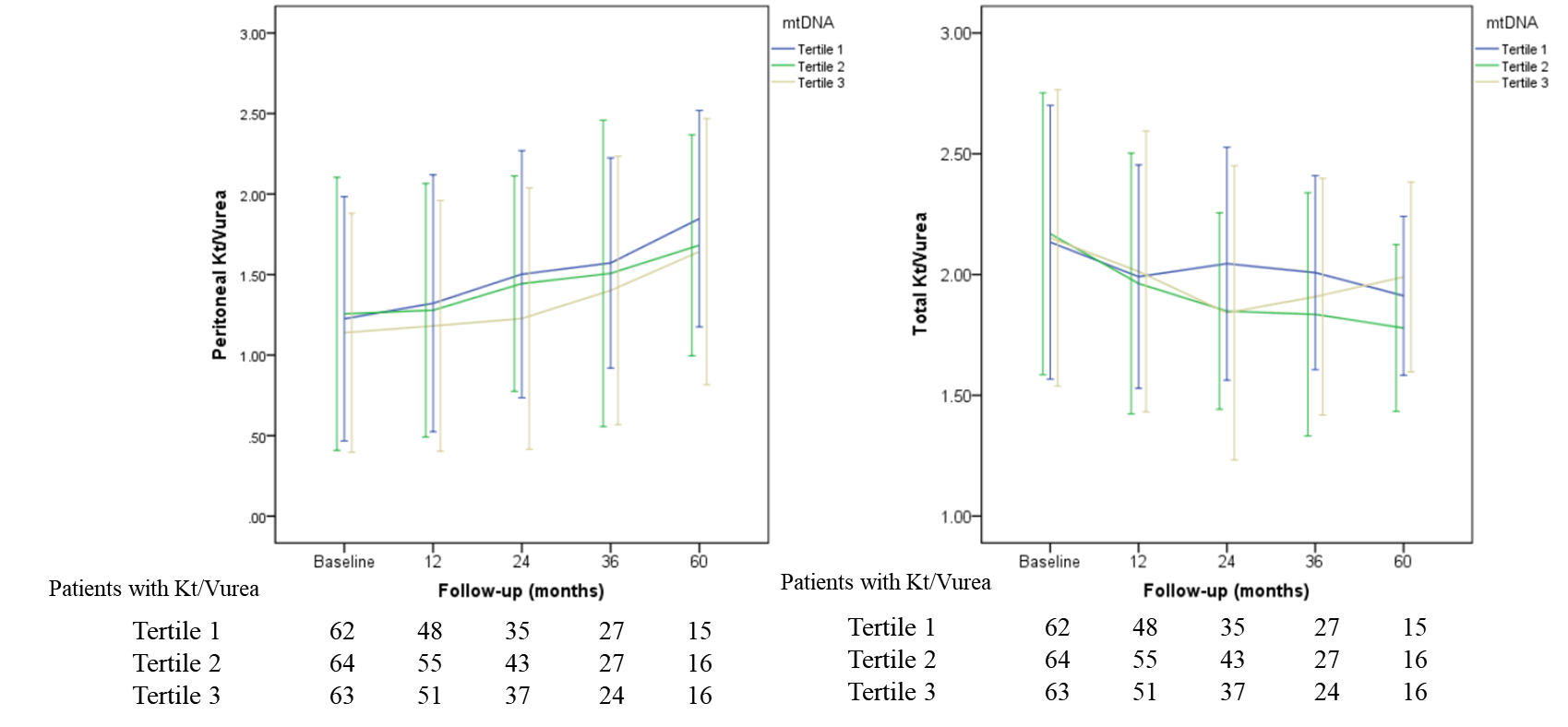


**Table S1**. Predictors of IL-6 (univariate analysis)

| Variable | Coefficient (95% CI) | *P* Value |
| --- | --- | --- |
| Age (per 10 years) | 0.016 (-0.002 to 0.033) | **0.076** |
| Sex (Female) | -0.060 (-0.106 to -0.014) | **0.011** |
| BMI (per 1 kg/m^2^) | -0.007 (-0.014 to 0.001) | **0.093** |
| CCI (per 1 point) | 0.007 (-0.011 to 0.025) | 0.426 |
| Diabetes | 0.010 (-0.061 to 0.082) | 0.773 |
| 2.5% glucose solution use | 0.004 (-0.043 to 0.051) | 0.868 |
| Dialysate glucose exposure ( per 10 g) | <0.001 (-0.007 to 0.007) | 0.931 |
| Dialysate volume (per 1 L) | <0.001 (-0.017 to 0.016) | 0.964 |
| Urine volume (per 1 L) | 0.017 (-0.030 to 0.064) | 0.473 |
| CRP (per 10mg/ L) | 0.009 (-0.030 to 0.049) | 0.645 |
| Residual GFR (per 1ml/min) | 0.003 (-0.007 to 0.012) | 0.569 |
| Serum albumin (per 1g/dL) | -0.041 (-0.084 to 0.002) | **0.059** |
| Hemoglobin (per 1g/dL) | -0.005 (-0.019 to 0.009) | 0.505 |
| Time of PET (> 3 months) | 0.014 (-0.033 to 0.061) | 0.566 |
| Peritoneal Kt/Vurea (per 1 point) | 0.032 (-0.020 to 0.085) | 0.304 |
| Total Kt/Vurea (per 1 point) | 0.022 (-0.020 to 0.064) | 0.777 |
| mtDNA | 0.202 (0.160 to 0.244) | **<0.001** |
| IL-17A | 0.456 (0.110 to 0.803) | **0.010** |
| TNF-α | 0.669 (0.535 to 0.803) | **<0.001** |
| IFN-γ | 0.611 (0.405 to 0.816) | **<0.001** |

Abbreviations: BMI, body mass index; CCI, Charlson comorbidity index; CRP, C-reactive protein; Residual GFR, residual glomerular filtration rate; PSTR, Peritoneal solute transport rate; PET, Peritoneal equilibration test. mtDNA and cytokine coefficients are per log_10_ changes in concentrations.

**Table S2**. Predictors of PSTR (univariate analysis)

| Variable | Coefficient (95% CI) | *P* Value |
| --- | --- | --- |
| Age (per 10 years) | 0.009 (-0.119 to 0.137) | 0.891 |
| Sex (Female) | -0.515 (-0.853 to -0.177) | **0.003** |
| BMI (per 1 kg/m^2^) | -0.027 (-0.085 to 0.031) | 0.359 |
| CCI (per 1 point) | -0.072 (-0.205 to 0.061) | 0.287 |
| Diabetes | 0.020 (-0.505 to 0.545) | 0.940 |
| 2.5% glucose solution use | 0.590 (0.257 to 0.923) | **0.001** |
| Dialysate glucose exposure ( per 10 g) | 0.078 (0.026 to 0.129) | **0.003** |
| Dialysate volume (per 1 L) | 0.168 (0.048 to 0.288) | **0.006** |
| Urine volume (per 1 L) | -0.141 (-0.484 to 0.201) | 0.419 |
| Time of PET (> 3 months) | -0.130 (-0.474 to 0.214) | 0.459 |
| Residual GFR (per 1 ml/min) | -0.015 (-0.084 to 0.053) | 0.665 |
| Albumin (per 1 g/dL) | -0.108 (-0.427 to 0.210) | 0.505 |
| Hemoglobin (per 1 g/dL) | -0.052 (-0.156 to 0.052) | 0.330 |
| CRP (per 10 mg/L) | 0.009 (-0.020 to 0.038) | 0.545 |
| Peritoneal Kt/Vurea (per 1 point) | -0.020 (-0.059 to 0.018) | 0.304 |
| Total Kt/Vurea (per 1 point) | 0.004 (-0.027 to 0.036) | 0.777 |
| mtDNA | 1.208 (0.877 to 1.539) | **<0.001** |
| IL-6 | 3.676 (2.767 to 4.584) | **<0.001** |
| IL-17A | 0.494 (-2.097 to 3.086) | 0.708 |
| TNF-α | 2.511 (1.355 to 3.666) | **<0.001** |
| IFN-γ | 3.356 (1.783 to 4.928) | **<0.001** |

Abbreviations: BMI, body mass index; CCI, Charlson comorbidity index; hs-CRP, high sensitivity C-reactive protein; Residual GFR, residual glomerular filtration rate; PSTR, Peritoneal solute transport rate; PET, Peritoneal equilibration test. mtDNA and cytokine coefficients are per log_10_ changes in concentrations.

**Table S3.** Risk factors of mortality (multivariable Cox regression model)

| Variable | Hazard Ratio (95% CI) | *P* Value |
| --- | --- | --- |
| Age (per 10 years) | 1.593 (1.163-2.183) | **0.004** |
| CCI (per 1 point) | 1.644 (1.057-2.558) | **0.028** |
| Urine volume (per 1L) | 0.221 (0.078-0.629) | **0.005** |
| Albumin (per 1 g/dL) | 0.485 (0.194-1.214) | 0.122 |
| Hemoglobin (per 1 g/dL) | 0.665 (0.529-0.838) | **0.001** |
| hs-CRP(per 10mg/L) | 1.080 (0.609-1.915) | 0.792 |
| mtDNA | 2.453 (0.726-6.649) | 0.118 |
| TNF-α | 0.071 (0.011-1.583) | 0.102 |

Abbreviations: CCI, Charlson comorbidity index; hs-CRP, high sensitivity C-reactive protein. mtDNA and TNF-α are per log_10_ changes in concentrations.
